# MannDB – A microbial database of automated protein sequence analyses and evidence integration for protein characterization

**DOI:** 10.1186/1471-2105-7-459

**Published:** 2006-10-17

**Authors:** Carol L Ecale Zhou, Marisa W Lam, Jason R Smith, Adam T Zemla, Matthew D Dyer, Thomas A Kuczmarski, Elizabeth A Vitalis, Thomas R Slezak

**Affiliations:** 1 Lawrence Livermore National Laboratory, Pathogen Bio-informatics, Livermore, CA, USA; 2 Virginia Bioinformatics Institute, Virginia Polytechnic Institute and State University, Blacksburg, VA, USA

## Abstract

**Background:**

MannDB was created to meet a need for rapid, comprehensive automated protein sequence analyses to support selection of proteins suitable as targets for driving the development of reagents for pathogen or protein toxin detection. Because a large number of open-source tools were needed, it was necessary to produce a software system to scale the computations for whole-proteome analysis. Thus, we built a fully automated system for executing software tools and for storage, integration, and display of automated protein sequence analysis and annotation data.

**Description:**

MannDB is a relational database that organizes data resulting from fully automated, high-throughput protein-sequence analyses using open-source tools. Types of analyses provided include predictions of cleavage, chemical properties, classification, features, functional assignment, post-translational modifications, motifs, antigenicity, and secondary structure. Proteomes (lists of hypothetical and known proteins) are downloaded and parsed from Genbank and then inserted into MannDB, and annotations from SwissProt are downloaded when identifiers are found in the Genbank entry or when identical sequences are identified. Currently 36 open-source tools are run against MannDB protein sequences either on local systems or by means of batch submission to external servers. In addition, BLAST against protein entries in MvirDB, our database of microbial virulence factors, is performed. A web client browser enables viewing of computational results and downloaded annotations, and a query tool enables structured and free-text search capabilities. When available, links to external databases, including MvirDB, are provided. MannDB contains whole-proteome analyses for at least one representative organism from each category of biological threat organism listed by APHIS, CDC, HHS, NIAID, USDA, USFDA, and WHO.

**Conclusion:**

MannDB comprises a large number of genomes and comprehensive protein sequence analyses representing organisms listed as high-priority agents on the websites of several governmental organizations concerned with bio-terrorism. MannDB provides the user with a BLAST interface for comparison of native and non-native sequences and a query tool for conveniently selecting proteins of interest. In addition, the user has access to a web-based browser that compiles comprehensive and extensive reports. Access to MannDB is freely available at .

## Background

MannDB was created to meet a need for rapid, comprehensive sequence analysis with an emphasis on protein processing, surface characteristics, and functional classification to support selection of pathogen or virulence-associated proteins suitable as targets for driving the development of protein-based reagents (e.g., antibodies, non-natural amino-acid ligands, synthetic high-affinity ligands) for pathogen detection [1,2]. Because comprehensive analyses of this type required using a large number of open-source tools, and because it was necessary to scale the computations for analysis of whole proteomes, we built a fully automated system for executing sequence analysis tools and for storage, integration, and display of protein sequence analysis and annotation data. In order to be able to rapidly examine and compare whole bacterial and viral proteomes for selection of suitable target proteins for bio-defense applications, we compiled data for whole proteomes from representative organisms from all categories of biological threat agents listed by several governmental agencies: APHIS, CDC, HHS, USDA, USFDA, NIAID, and WHO [3-9] as well as taxonomic near-neighbor species as appropriate. Therefore, the scope of MannDB is automated sequence analysis and evidence integration for proteins from all currently recognized bio-threat pathogens. Emphasis is placed upon analyses that are most useful in characterizing potential protein targets and surface motifs that could be exploited for development of detection reagents. The content of MannDB is updated on a regular basis.

In recent years several software systems and accompanying databases have been developed for microbial genome annotation, each with a particular emphasis [10-19]. Some databases place an emphasis on gene prediction and DNA-based analyses vs. protein sequence-based analyses, or provide automated (primary) vs. curated (secondary) annotations. Although microbial annotation databases frequently include predictions of biological, chemical, structural, and physical properties of proteins (e.g., antigenicity, post-translational modifications, hydrophobicity, membrane helices), none currently offers the comprehensive suite of analyses (see MannDB website for complete list of tools) contained within MannDB for characterizing viral as well as bacterial proteins from human and agricultural/veterinary pathogens of interest to the bio-defense community and for rapidly identifying putative virulence-associated proteins for development of functional assays. The MannDB database was built and linked to MvirDB [20] in order to meet these requirements. In addition, we focus on sequence analyses that assist in selection of protein features (e.g., surface characteristics) most suited for targeting detection reagent development.

## Construction and content

**Figure 1:**
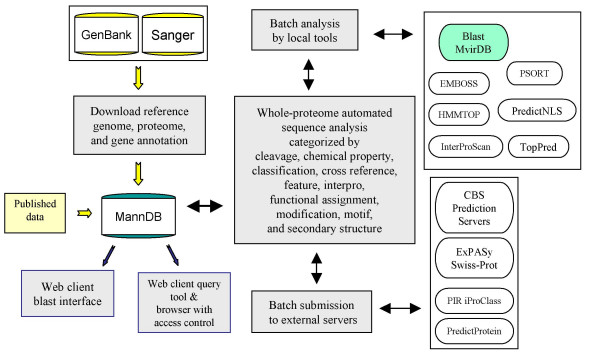
Data flow diagram for MannDB sequence analysis pipeline. External data sources (yellow) are downloaded into MannDB. Software systems (lavender boxes) process and enable display of data. MannDB pipeline manager controls execution of open-source tools (ovals) and blast against MvirDB (green oval).

MannDB is implemented as an Oracle 10 g relational database. The schema for MannDB data organization is available on the website. MannDB captures results from our fully automated, high-throughput, whole-proteome sequence analysis process pipeline, depicted in Fig. 1. Proteomes (lists of hypothetical and known proteins) representing human bacterial and viral pathogens and near-neighbor species are downloaded from GenBank and parsed into MannDB. Whenever possible, we begin with gene calls on finished genomes. However, the system can be used to predict genes on draft genomes, and can be used to analyze arbitrary lists of protein sequences. Reference genomes are updated on a quarterly basis to ensure that the software tools are being run on current sequence data. Annotations from SwissProt are downloaded when GenBank entries contain SwissProt identifiers, or when identical sequences are detected by blasting MannDB entries against the SwissProt protein fasta database. MannDB contains at least one reference genome for each category of pathogen listed as a bio-threat organism on websites maintained by APHIS, CDC, HHS, USDA, USFDA, NIAID, and WHO. Open-source tools are run either on local systems or by means of batch submission to external servers. As of this writing the system executes 36 tools, which are listed on the MannDB web site. Automated sequence analyses include predictions of post-translational modifications, structural conformation, chemical properties, functional assignment, and antigenicity, as well as motif detection and pre-computed BLAST against protein and nucleic acid sequences in MvirDB, our database of microbial virulence factors, protein toxins, and antibiotic resistance genes [20]. Tools that are run in-house are updated periodically to ensure that the system is running the most recent software versions against the most recent data sets. Tools are selected and input parameters are set according to the taxon of the organism from which the protein set is constructed. For example, some tools (e.g., NetPicoRNA; [21]) are run only on specific organisms, whereas others (e.g., SignalP; [22]) have taxon-specific settings. In some cases we run more than one tool for a similar prediction. TMHMM and TopPred both predict membrane helices, but results may differ, for example, in the start and end residues for a given segment. Our strategy is to employ more than one tool, when available, so that conflicting results can be noted and evaluated by the user. In parsing results from each tool, data are inserted into one of nine tables (see schema on web site) depending on the type of prediction (e.g., protein chemistry); tools that make similar predictions tend to produce similarly structured output (although formatting differs considerably), which facilitates data storage and retrieval.

**Figure 2:**
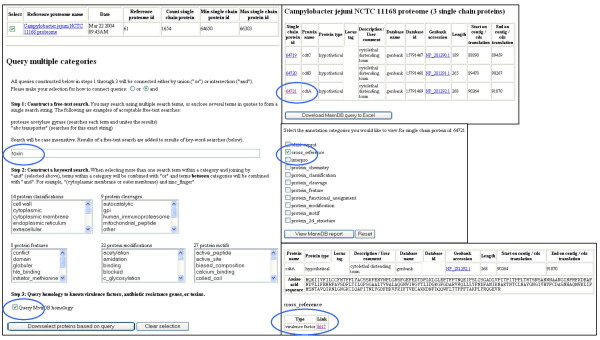
MannDB database query and browser sample web pages. In this example, user has selected the *Campylobacter jejuni *proteome (left), entered free text "toxin" (top oval), and checked the MvirDB homology checkbox (bottom oval), resulting in 3 database hits (top right). Selecting single chain protein id 64721 (top right, oval), followed by the "cross-reference" checkbox (middle right, oval) brings up a report page (bottom right) displaying the MvirDB cross reference link (oval).

A web client browser enables viewing of automated analysis results, annotations, and links to MvirDB (Fig. 2). The user first selects a proteome, then a specific protein for which to view summary results, and finally selects the specific categories of analysis to be viewed. Only analyses returning results are displayed. Hyperlinks to external data sources are provided for additional information whenever external database identifiers are returned. The MannDB toolset includes a BLAST interface, which can be used to quickly identify an entry of interest by its sequence, when the gene name or locus tag is unknown, or to identify protein sequences related to a sequence of interest. A query tool allows the user to construct 3 types of searches: 1) free-text searches against all database fields that contain descriptive information, including fields containing gene names or external database identifiers, 2) structured searches against specific analysis types, and 3) a search for proteins linked to entries in MvirDB either by common unique identifier or by pre-computed blast homology. Reports and results sets from the query tool can be downloaded into Excel.

## Utility and discussion

MannDB provides users with pre-computed sequence analyses for complete proteomes of bacterial and viral pathogens from several governmental agencies' lists of bio-threat agents. The genomes and tools are maintained up to date, with predictions being re-run every 3 months. The user can browse proteomes, or can blast sequences against MannDB to pull up related entries and associated data. MannDB provides a convenient source of automated sequence analyses and downloaded annotation information for whole proteomes of human pathogenic bacteria and viruses and has a high degree of integration with external databases.

MannDB provides sequence analysis information of primary interest to researchers in the bio-defense community. We have been using MannDB for several years to "annotate" DNA signatures [1] and more recently to assist collaborators in efforts to down-select from whole bacterial and viral genomes to identify suitable protein targets and protein features for driving the development of detection reagents [2]. For example, a common requirement for a detection assay is that it be performed with minimal sample disruption. Therefore, an initial down selection for proteins expected to be on the surface of a bacterial particle might entail identification of proteins that are predicted to be secreted or membrane bound by using tools such as PSORT [23,24], TMHMM [25], SignalP, TargetP [26], TopPred [27], and HMMTOP [28]. Having results from several tools that provide similar predictions but using different algorithms or slightly different approaches allows us to compare predictions and make selections with greater confidence. Identification of surface features for targeting of detection reagents is done primarily by means of additional sequence- and structure-based analyses [2], although predictions pertaining to post-translational modifications (e.g., glycosylation, cleavage) are taken into consideration as they may affect protein recognition.

## Conclusion

MannDB is a genome-centric database containing comprehensive automated sequence analysis predictions for protein sequences from organisms of interest to the bio-defense research community. Computational tools for the MannDB automated pipeline were selected based on customer needs in providing down selections from large sets of proteins (e.g., whole proteomes) to short lists of proteins most suitable for developing reagents to be used in field assays for detection of pathogens. For that reason we have focused our efforts on applying tools that would enable selection of proteins that meet assay requirements, such as cellular localization, that would assist in determining the value of a surface feature for targeting ligand binding, or that would identify antigenic sub-sequences of particular value in antibody development. As the goals of some of these assays have been to detect toxins or proteins associated with virulence, we constructed hard links between protein sequences in MannDB with entries in MvirDB in order to conveniently identify and characterize protein targets and features for these applications. We believe that MannDB will be of general use to the bio-defense and medical research communities as a resource for predictive sequence analyses and virulence information.

## Availability and requirements

MannDB is freely accessible at . Although the software that populates and updates MannDB is not open-source, the user may request collaborative sequence analysis services by contacting ppi_group@kpath.llnl.gov.

## List of abbreviations

BLAST. Basic local alignment search tool.

APHIS. Animal and Plant Health Inspection Service.

CDC. Centers for Disease Control and Prevention.

HHS. Health and Human Services.

USDA. United States Department of Agriculture.

USFDA. United States Food and Drug Administration.

NIAID. National Institute of Allergies and Infectious Diseases.

WHO. World Health Organization.

## Authors' contributions

CEZ and ML designed the database with input from TK. CEZ and ML designed and ML and TK built the pipeline and web interface tools. MDD and JS built software modules to facilitate porting of the data and interfaces beyond the LLNL firewall. AZ and BV contributed to design concepts for applications to protein-based studies. TS initiated and CEZ designed and managed the project. All authors read and approved the final manuscript.
